# Development of a real-world simulated instrument for evaluating visuospatial working memory: a preliminary psychometric study on older adults

**DOI:** 10.1186/s12877-024-05140-9

**Published:** 2024-06-24

**Authors:** Zahra Mirchi, Mohammad Taghi Kheirkhah, Reza Khosrowabadi, Javad Salehi Fadardi, Mojdeh Ramezani

**Affiliations:** 1 Faculty of Education and Psychology, Ferdowsi University of Mashhad, Mashhad, Iran; 2 Institute for Cognitive and Brain Sciences, Shahid Beheshti University, no 18., Daneshjoo Blvd., Shahid Shahriari Sq., Tehran, Iran; 3 School of Community and Global Health, Claremont Graduate University, Claremont, CA USA; 4 School of Psychology, Bangor University, Bangor, UK; 5 Faculty of Psychology and Educational Sciences, University of Tehran, Tehran, Iran

**Keywords:** Visuospatial working memory, Elderly people, Ecological validity, Psychometric properties

## Abstract

**Background:**

A prevalent challenge in neuropsychological assessment, particularly when utilizing instruments designed for controlled laboratory environments, is that the outcomes may not correspond to an individual’s real-life status. Accordingly, assessments of visuospatial working memory (VSWM) conducted in such settings might fail to capture certain facets of this function, as it operates in real life. On the other hand, entirely ecological assessments may risk compromising internal validity. This study aimed to develop an intermediate mode of assessment that measures VSWM in older adults by employing a setting, a task, and a response format that aligns closely with both laboratory and ecological assessments. Furthermore, a preliminary investigation was carried out to study the variations in spatial cognition among different demographic groups.

**Methods:**

In a two-session study, 77 healthy older adults, eight patients with mild cognitive impairment (MCI), and seven patients with Alzheimer’s disease (AD) were recruited to complete the wayfinding questionnaire (WQ), the Corsi block-tapping task (CBTT), and the Spatial Memory Table (SMT). The SMT is a novel instrument developed specifically for this study, aiming to provide a more accurate measure of VSWM performance in older adults’ everyday life. Test-retest and split-half reliabilities, as well as the face, content, concurrent, convergent, and known-groups validities, were analyzed to investigate the psychometric properties of the SMT.

**Results:**

The analyses were mainly centered on studying the psychometric properties of the SMT. Test-retest reliability (*r* = .753, *p* < .001) and split-half reliability (*ρ*SC = 0.747) were found to be acceptable. Concurrent validity using CBTT (*r* = .264, *p* = .021), convergent validity using WQ subscales (navigation and orientation: *r* = .282, *p* = .014; distance estimation: *r* = .261, *p* = .024), and known-groups validity using the SMT scores among people with MCI and AD (*χ2* = 35.194, *df* = 2, *p* < .001) were also indicative of the instrument’s good validity. Data analysis also revealed acceptable levels of face validity (*U* = 4.50; *p* = .095) and content validity (CVR ≥ 0.60). As a result of comparing VSWM and wayfinding variables across genders and education levels, a significant difference was observed for navigation and orientation and spatial anxiety between women and men (*p* < .05). None of the variables were different among education levels.

**Conclusion:**

The SMT was found to be a reliable and valid tool for measuring VSWM performance in older adults. Given these findings, the SMT can be regarded as a measure that sufficiently approximates both laboratory and real-life demands for VSWM. Additionally, the instrument demonstrated a preliminary acceptable capacity to differentiate between healthy individuals and those with MCI and AD.

**Supplementary Information:**

The online version contains supplementary material available at 10.1186/s12877-024-05140-9.

## Introduction

There is a long history of considering ecology in behavioral studies [1, 2]. However, recently, ecological validity has received special attention in neuropsychology [3–5]. Ecological validity is the extent to which the findings of a research design/measurement predict behaviors in real-life situations so that the findings both represent it and can be generalized to it [6].

One of the challenges with neuropsychological assessment by tools developed for use in controlled laboratory settings is that the outcomes may not correspond to the individual’s status in real life (lack of veridicality; [7]). This discrepancy arises because real-life settings, stimuli, and responses vary from those designed under controlled conditions [8]. Additionally, while in real life, an individual is surrounded by a multitude of different environmental characteristics [9], under laboratory settings, this important aspect, the ecosystem, is overlooked. Numerous studies have examined the correlation between the results of standard cognitive tests and real-life demands. In many cases, what is measured in the laboratory is roughly distinct from what occurs in real life [10–13].

Domains such as spatial and social cognition are strongly tied to one’s activities within the environment [14]. Therefore, knowing where and who we (and others) are is not something easy to study by limiting the environment. It has been shown that there is only a moderate correlation between what standard neuropsychological tests measure and individuals’ complaints of memory in everyday life [15, 16]. The distinction between complaining about what disrupts daily life functions and what can be measured in the laboratory suggests the need for standard tools to measure real-life functions, and the greater the gap is, the greater the need for ecological assessment.

Visuospatial working memory is a form of memory through which one can temporarily store information about places and spatial relationships, perform actions, and be oriented in the environment. This cognitive function is essential for activities such as wayfinding [17]. Due to its dependence on the characteristics of the environment, the need for ecological assessments of spatial memory is even more crucial than for assessments of other cognitive functions [18]. Nadolne and Stringer showed that there is no significant correlation between the results of classical neuropsychological tests and the ability to navigate in real life [19]. There is evidence suggesting that if people (i.e., children, younger adults, and older adults) engage in movement while performing a laboratory VSWM task, their performance in VSWM (both encoding and recall) decreases [20]. This shows that laboratory results may not always take into account a key component of spatial cognition, which is orienting in the environment [21]. Theoretically, VSWM and other processes of spatial cognition (e.g., mental rotation, spatial navigation, processing depth and motion) are interconnected tasks in real life [22–24].

The investigation of VSWM in older adults is of paramount importance due to its implications for cognitive aging. As individuals age, working memory performance declines, with VSWM being particularly age-sensitive [25]. This decline in VSWM can impact spatial orientation, a crucial ability for daily activities such as navigation [26]. Furthermore, both verbal and Visuospatial working memory measures uniquely predict reading and math measures, suggesting that a domain-general working memory system contributes to performance on intelligence measures [27]. The functional architecture of the visuospatial sketchpad, which may be affected by aging, is another area of interest. Differential visual and/or spatial interference effects may be observed across younger and older adults as a consequence of different processing abilities [28]. On the other hand, studies have pointed out the ability of visuospatial memory to differentiate healthy elderly individuals from neurogenerative and MCI patients [29]. Accordingly, VSWM measurement can be a priority in investigating the underlying mechanisms of older adults’ cognition and monitoring of cognitive aging. Such instruments would provide a clearer representation of an individual’s cognitive abilities in real-world contexts, thereby informing interventions to support cognitive health in older adults more effectively. Thus, the investigation of methods for measuring VSWM in older adults is not only important but also necessary for advancing our understanding of cognitive aging and developing effective interventions.

Most laboratory neuropsychological tests for measuring VSWM, instead of focusing on the direction of objects, which is a key feature in defining space, simply refer to their location on the display [30]. At the same time, some of the visual attributes of an object, including its shape and color, which are integral properties for encoding and retrieving spatial information, are not always found to contribute [31, 32]. In this regard [33], showed that memory for locations interacts with memory for shapes and colors. Therefore, these subsystems are dependent on each other. This finding contradicts the classical hypothesis of independence of VSWM subsystems [34]. In addition, the lack of sufficient evidence regarding the veridicality of neuropsychological assessment methods for VSWM highlights the need to develop new methods that are more representative of real-world demands. Although ecological assessments may satisfy the demands of the environment, it is worth noting that they always have limitations. Focusing on merely ecological assessment compromises the measurement’s internal validity. Most likely, neither or both intermediate modes of assessment would be more efficient methods for the assessment of VSWM. This study aimed to develop an intermediate mode of assessment that measures VSWM in older adults by employing a setting, task, and response format that aligns closely with both laboratory and ecological assessments.

The primary objective of the current study was to conduct a preliminary examination of the psychometric properties of the instrument developed in this study for the purpose of assessing VSWM in elderly individuals. This included evaluating its stability and consistency (reliability), as well as its face, content, concurrent, convergent, and known-groups validities. Given that differences in VSWM and wayfinding have been previously reported in studies as being subject to variations among demographic groups (gender and education level) [35–37], these differences were considered as a secondary objective in the present study.

## Methods

### Participants

For the present study, an a priori sample size was estimated using G*Power [38] to be *n* = 63 (*ρ* = 0.40; *α* = 0.05; 1 – *β* = 0.95; one-tailed; correlation: bivariate normal model). A total of 80 older adults were selected from the general population residing in Tehran based on convenience sampling and were included in the study. The participants had to be older than 65 years old and have normal or corrected-to-normal vision and hearing. To control for confounding variables associated with handedness, all participants were right-handed. They were not to have any permanent neurological diseases or psychiatric diagnoses. To be eligible for the study, it was necessary for participants to sign the informed consent form. In the process of data collection, one person was not included due to having previously experienced a traumatic brain injury, and the data of two participants were excluded due to missing or outlier responses (*n* = 77; 38 males; age *M* = 68.71; age *SD* = 3.98). The dropout rate was greater for the second session of the study. In the second session, 24 participants refused to participate. The present study also included seven participants with AD (3 females; age *M* = 72.32; age *SD* = 4.59) and eight participants with MCI (3 females; age *M* = 70.64; age *SD* = 4.26) who were diagnosed by a specialist and lived with a partner. These individuals were recruited using an online invitation. The inclusion criteria for these groups were identical to those for the healthy group, with the requirement that individuals in these groups must have received a clinical diagnosis of AD or MCI.

### Materials

#### Wayfinding questionnaire

The Wayfinding Questionnaire (WQ) is considered a reliable and valid instrument proposed by De Rooij and colleagues and contains 22 items in 3 subscales, namely, navigation and orientation (NO; 11 items), distance estimation (DE; 3 items), and spatial anxiety (SA; 8 items), with scores ranging from 1 to 7 (*1 = not at all applicable to me* and *7 = fully applicable to me;* [39]). A lower score on each subscale indicates more wayfinding complaints. Each subscale represents a different aspect of wayfinding and is not combined in one total score.

#### Corsi block-tapping task

The Corsi block-tapping task (CBTT), designed and utilized in the early 1970s, serves as a measure of visuospatial working memory [40]. This test, like other tests related to working memory, measures the number of items that can be retained and recalled (memory span). In this study, a standard computerized version of the task, which is available in The Psychology Experiment Building Language (PEBL) [41], was used. Nine blocks were displayed on a 17-inch computer screen, and the participant was required to remember the number and sequence of the illuminated blocks in each trial and respond by selecting the blocks in sequence. Only the forward sequence of stimuli was followed in the task. If the response was correct, the number of illuminated blocks would increase in the subsequent trials. The test continued until the participant was unable to correctly select the blocks in the same order as when they were illuminated. Although normative data for CBTT exist [42], in the current study, the performance in the task was used solely as a criterion for validity. The task was administered on a standard desktop computer running a Windows operating system. To ensure that the timing and presentation of the stimuli remained consistent across all trials and participants, a computer with a high configuration was used. The participant was positioned approximately 60 cm away from the screen.

#### Spatial memory table: development and evaluation

**Fig. 1:**
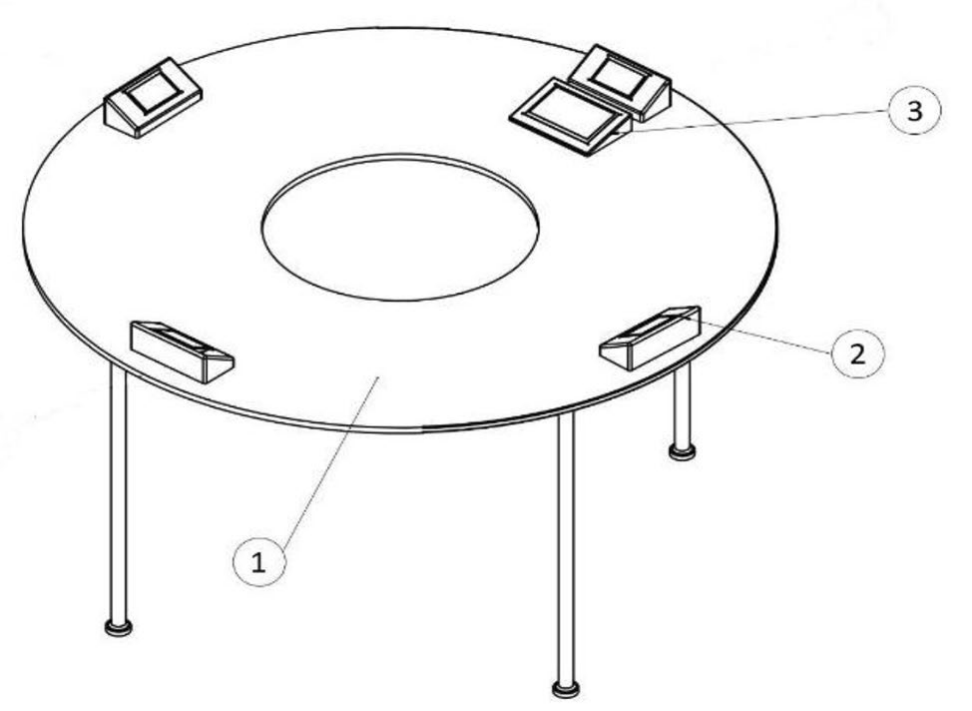
SMT and its main components. (1) surface and legs which keep the rest of the components stable, (2) 4.3 inches displays, buzzer, Mega Shield, and Arduino board, (3) 7 inches touch screen dynamic keyboard, CPU, and keyboard and CPU holder

**Fig. 2:**
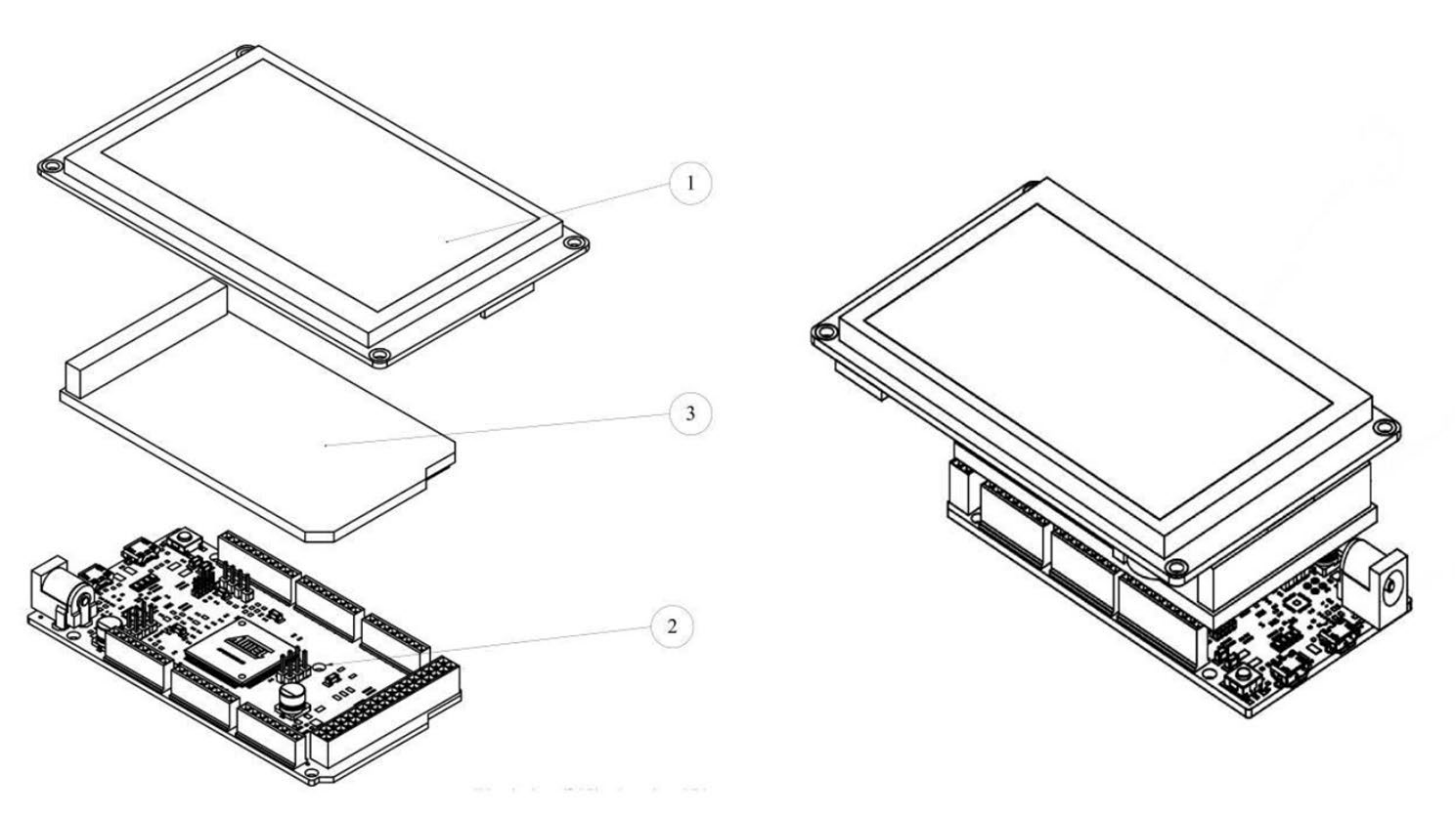
Microcontroller set. (1) 4.3-inch display, (2) Arduino mega 2560 rev3, (3) Arduino Mega Proto Shield Rev3 (PCB)

To measure VSWM, a donut-shaped table (*r* = 75 cm; *h* = 90 cm) was used, on which there were four 4.3-inch displays equipped with buzzers and a 7-inch touch screen dynamic keyboard to record the responses. As shown in Fig. 1, there was a space for the participant to stand in the middle part of the table. The stimuli were presented by the command of a microcontroller (Fig. 2). During each trial, the processor randomly determined which screens should be illuminated, in what order, and what stimulus was presented on the display (circle, square, triangle, or star in red, yellow, green, or blue). Simultaneously, spatial audio cues were played by the buzzer connected to the screens to determine the order in which the participants should turn in different directions. In each direction, they face a stimulus to memorize. The stimuli remained on the display for three seconds. After participants turned in the direction to remember the pattern of stimuli, they returned to the initial location of the test to input their response. The response consisted of a pattern of stimuli that, depending on the task level, included one shape and one color (for example, a red circle) in various directions or multiple shapes and colors in various directions. A correct answer was considered when the participant could accurately report all the stimuli in the same order as presented, with matching colors and shapes. The responses were stored in the storge of the instrument and could be retrieved later. In this study, similar to the CBTT, the answers were recorded in a forward manner, and the participant’s score comprised the sum of all their successful attempts. If the last successful attempt included both a failure and a success, the final score was equal to the sum of the previous successful attempts plus half.

The instrument was patented in the Real Estate Registration Organization of Iran under the name Method for Assessment and Enhancement of Visuospatial Memory Performance (Classification: A61B 5/16; IR101591).

To assess the face and content validity of the spatial memory table (SMT), after designing and developing the instrument, 10 raters (i.e., five experts and five participants) were asked to evaluate the face validity, and five expert raters (the same ones) were asked to evaluate the content validity. As indicated in Table 1, the raters reported their level of agreement regarding the prepositions proposed in an 11-item questionnaire (5-point Likert scale), in which the first six and last five items were related to face and content validity, respectively. The sample from the general population answered only the first six items. The Mann-Whitney U test was used to compare the responses of the two groups of experts and the general population on all items related to the instrument’s face. The results showed that there was no significant difference between the two groups (*U* = 4.50; *p* = .095). Content validity was calculated utilizing the content validity ratio (CVR) and content validity index (CVI). For all of the items, the CVR was greater than the criterion ( [43]; CVR ≥ 0.60) and at an acceptable level. In addition, the CVI at the level of content-related items was at an acceptable level ( [44]; I-CVI ≥ 0.79). Furthermore, calculating the index of content validity at the scale level by applying the universal agreement method indicated the high content validity of the scale (S-CVI/UA ≥ 0.8). Thus, some evidence was collected about the face and content validity of the instrument before the study.

**Table 1:** Face and content validity questionnaire

**Face validity**					
		Experts (*n* = 5)	GP (*n* = 5)		
		M (SD)	M (SD)	Mann-Whitney *U*	*p* value
1	All of the individuals older than 7 years who are in appropriate physical health can apply this instrument.	4.20 (0.84)	4.00 (0.70)	10.50	0.690
2	Stimuli and procedures are regarded as appropriate for measuring Visuospatial working memory.	3.40 (0.55)	3.20 (1.10)	9.50	0.548
3	The stimuli simulate to those of laboratory methods.	4.40 (0.89)	3.00 (0.70)	3.00	0.056
4	The procedures simulate to those of laboratory methods.	3.80 (0.84)	3.60 (0.55)	11.00	0.841
5	The stimuli are close to those in real-life.	4.40 (0.84)	3.60 (0.89)	5.50	0.151
6	Procedures are related to real-life demand.	3.00 (0.54)	3.60 (1.34)	9.50	0.548
**Content validity**					
				Experts (*n* = 5)	
				CVR	I-CVI
7	The aspect related to spatial orientation is properly included in the instrument.			1.00	1.00
8	The phenological loop gets involved in the process in the instrument.			0.60	0.80
9	Visuospatial sketchpad gets involved in the process.			0.60	0.80
10	To provide the response, the information is required to be manipulated in the working memory.			0.60	0.80
11	All of the aspects related to Visuospatial working memory are measured using this instrument.			0.60	1.00

Items 1–6 evaluate face validity and items 7–11 evaluate content validity; 1 = strongly disagree and 5 = strongly agree; GP, general population; CVR, content validity ratio

### Procedure

This was a two-session study, with the second session designed solely to obtain SMT retest data from the healthy group. In the first session, the healthy participants were initially asked to perform the CBTT using a desktop computer, followed by completing the paper-pencil Wayfinding Questionnaire. Subsequently, their performance was recorded in the SMT. The unhealthy participants only participated in the SMT assessment. While using SMT, instructions were given verbally by the experimenter. Participants prepared for the task by standing in the space within the table. The participants were required to turn to the direction from which the buzzer was heard and to remember the presented stimulus from the display in that direction. In the following, they were required to return to the point where the test began and enter their response—i.e., the type and sequence of stimuli presented. The entire process was regarded as a single trial followed by subsequent similar trials if successfully completed. As the trials approached the end, the number of stimuli presented increased (up to nine). If they failed, another trial with the same number of stimuli but different types and sequences was presented. Two consecutive failures in a single trial meant the termination of the test, and the number of successful trials was considered the individual’s overall performance score in the test. To ensure that participants understood the instructions, they were required to participate in two practice trials with a minimum number of stimuli. After the mentioned process was completed, all participants (except for participants with AD and MCI) were asked to return to participate in the second data collection session within one week. The entire data collection process took about 45 min for the first session and about 20 min for the second session.

### Data analysis

Pearson’s correlation coefficient between two different measurement occasions was calculated to examine test-retest reliability, and the split-half coefficient was calculated to investigate the instrument’s consistency following the method introduced by Steinke and Kopp [45]. To check concurrent validity and convergent validity, Pearson’s correlation coefficient (one-tailed) was calculated, and to analyze the known-groups validity, Kruskal–Wallis one-way analysis of variance was used. The CBTT, a recognized valid tool for VSWM, was used to investigate concurrent validity. The WQ subscales (i.e., NO and DE) were employed as measures of convergent constructs to VSWM for convergent validity. Considering that the SA subscale of the WQ did not have a functional aspect, it was not possible to draw a logical connection between this construct and the performance in VSWM. However, the data from this subscale were incorporated into subsequent analyses. Eventually, the performance in SMT among people with MCI and AD was utilized to investigate known-groups validity.

As a secondary objective, response variables including VSWM (measured by SMT), VSWM (measured by CBTT), and NO, DE, and SA (measured by the Wayfinding Questionnaire), were compared across two gender subsamples of men and women and three educational levels, employing nonparametric analyses (i.e., Mann–Whitney U test and Kruskal–Wallis test) separately.

## Results

### Sample characteristics

Table 2 shows the demographic characteristics of the participants, such as age, gender, education level, and history of neurological or psychiatric problems, as well as the means and standard deviations of the study variables.

**Table 2:** Descriptive characteristics of study sample

	Healthy		MCI		AD	
	*n* = 77	*M* (*SD*)/%	*n* = 8	*M* (*SD*)/%	*n* = 7	*M* (*SD*)/%
Gender						
Male	38	49.35	5	62.50	4	57.14
Female	39	50.65	3	37.50	3	42.85
Age		68.71 (3.98)		70.64 (4.26)		72.32 (4.59)
Handedness						
Right	77	100	8	100	7	100
Vision						
Normal	19	24.67	2	25	2	28.57
Corrected	58	75.32	6	75	5	71.42
Hearing						
Normal	58	75.32	5	62.50	3	42.85
Corrected	19	24.67	3	37.50	4	57.14
Education						
Diploma or below	53	68.83	5	62.50	4	57.14
Associate and bachelor	18	23.38	3	37.5	2	28.57
Postgraduate education	6	7.79	0	0	1	14.28
Psychiatric diagnosis^*^						
No	77	100				
Yes			8	100	7	100
CBTT^†^	77	5.20 (0.81)				
WQ^‡^	77	4.84 (0.28)				
NO^§^	77	4.72 (0.43)				
DE^¶^	77	5.04 (0.56)				
SA^#^	77	4.94 (0.28)				
SMT^**^						
Test	77	5.01 (0.68)		3.93 (0.49)		3.28 (0.48)
Retest	53	4.88 (0.89)				

* Including psychiatric disorders and neurological conditions; † total raw score on forward Corsi block-tapping task; ‡ weighted average score on Wayfinding Questionnaire subscales; § average score on navigation and orientation subscale; ¶ average score on distance estimation subscale; # average score on spatial anxiety subscale; ** total raw score on forward Spatial Memory Table

### Normality check

The standardized skewness and kurtosis indices (statistic divided by standard error) were calculated for each response variable to evaluate the normality of their distributions. For all measurements, the *z* scores for skewness and kurtosis fell within the range of -1.96 and + 1.96. This indicates that the distributions did not significantly deviate from normality.

### Stability and consistency

**Fig. 3:**
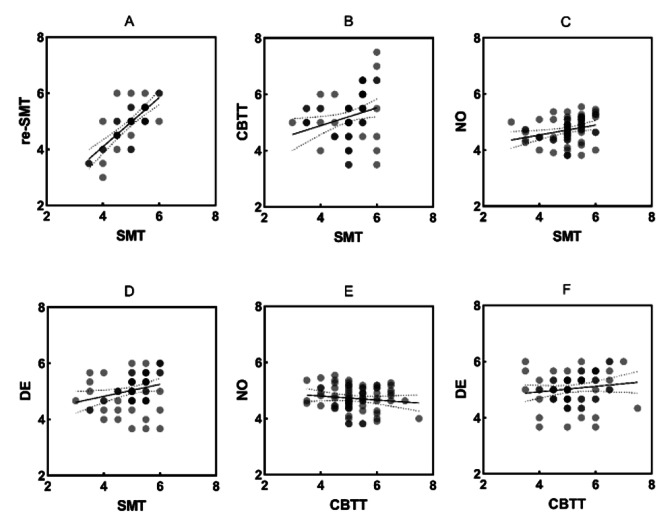
Correlations of the SMT, its retest, CBTT, and WQ dimensions

As illustrated in Fig. 3.A, test-retest reliability was obtained by Pearson’s correlation for the two SMT measurements for 53 participants (*n* = 24; did not participate in the retest session; 25 males; *r* = .753, *p* < .001). To investigate the consistency of the SMT, the split-half reliability (Angoff–Feldt coefficient) was calculated. Split-half reliability sampling utilizing 29 iterations revealed a median reliability coefficient of *ρ*_SC_ = 0.747. 95% of the sampled reliability coefficients were between *ρ*_SC_ = 0.676 and *ρ*_SC_ = 0.838. Based on the results, stability and consistency reliabilities were considered appropriate for the entire instrument.

### Concurrent, convergent, and known-groups validities

**Fig. 4:**
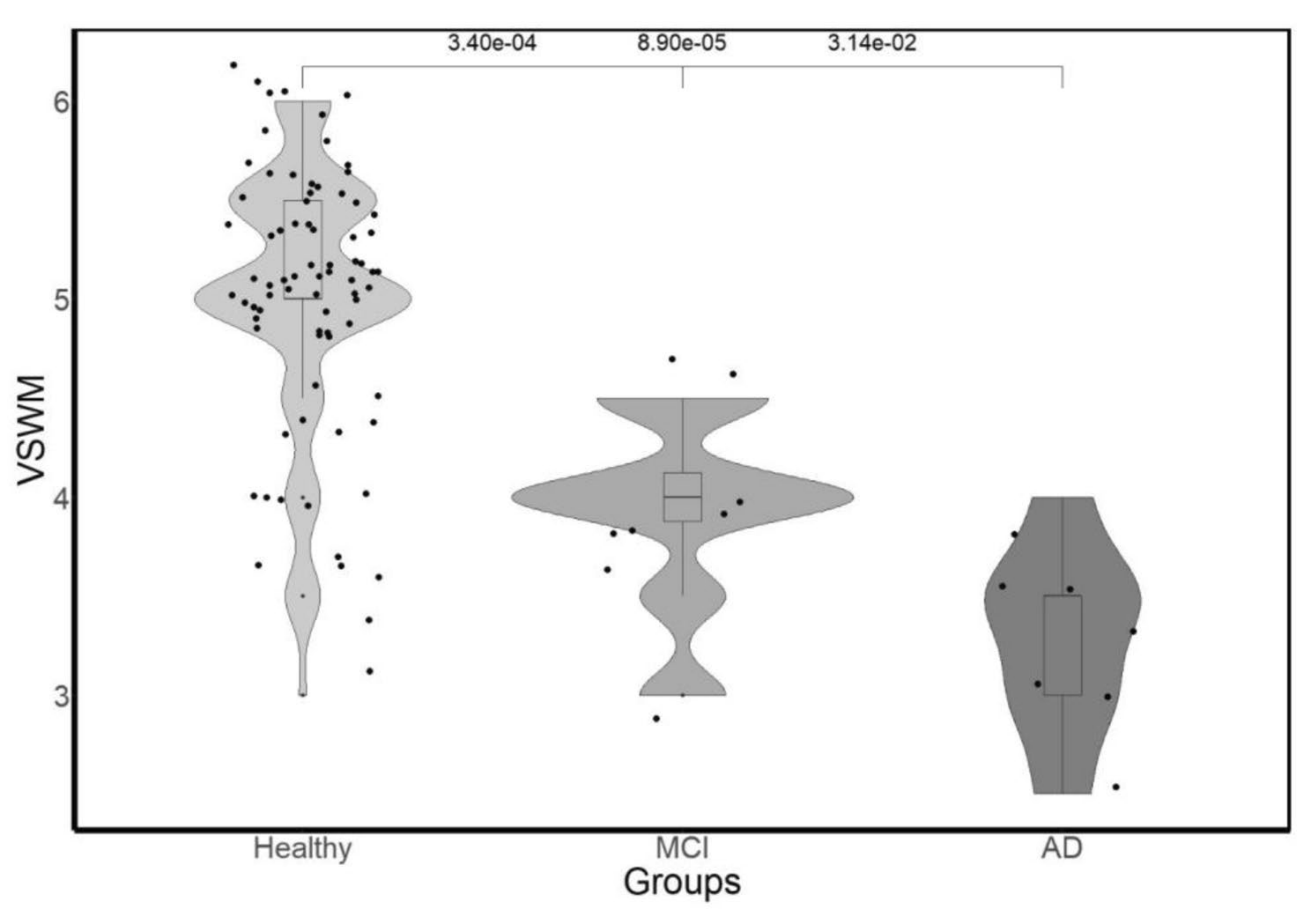
Violin plots representing the VSWM scores across three groups. Each plot illustrates the estimated density of VSWM scores within each group. The Healthy group shows a wider distribution around a VSWM score of 5, while the MCI and AD groups show more concentrated distributions around a VSWM score of 4 and 3.5, respectively

To investigate concurrent validity, Pearson’s correlation between CBTT and SMT scores indicated a significant positive correlation (*r* = .264, *p* = .021; Fig. 3.B). As demonstrated in Fig. 3.C-D, a significant positive relationship was observed between SMT and NO scores (*r* = .282, *p* = .014) and between SMT and DE scores (*r* = .261, *p* = .024; convergent validity), indicating that SMT, CBTT, and WQ measure the same or theoretically related constructs, respectively. Therefore, with its correlation with CBTT, the SMT is shown to be concurrently valid, while its convergent validity is demonstrated by its correlation with the WQ. One of these methods is applied in laboratory settings, and the other measures real-life demands. In addition, no correlation was reported between CBTT and wayfinding dimensions, while both CBTT and WQ were correlated with the SMT (Fig. 3.E-F). The results of the Kruskal‒Wallis one-way ANOVA on the performance in the SMT indicated a strong significant difference between the groups (*χ2* = 35.194, *df* = 2, *p* < .001; known-groups validity). The Wilcoxon rank-sum test was performed with *p*-value adjustment using the Holm method (Fig. 4).

### Variables across demographic groups

After conducting the analyses of reliability and validity, the investigation of potential differences in spatial memory variables across demographic groups could be viewed as a secondary objective. As the assumption of normality was not met in most instances (*p* > .05), this objective was pursued using nonparametric analyses. The variables SMT, CBTT, NO, DE, SA, and WQ were compared between men and women using Mann-Whitney U tests. These variables were also compared across three levels of education using Kruskal-Wallis tests. The results are summarized in Tables 3 and 4.

**Table 3:** Differences between males versus females on response variables

	Males	Females	U	Z	*p*	*r*
	MR	MR				
SMT	36.32	41.60	842.50	1.03	0.281	0.117
CBTT	38.88	39.11	745.50	0.04	0.967	0.005
NO	44.10	34.02	547	-1.97	0.048*	− 0.225
DE	40.11	37.91	698.50	-0.43	0.663	− 0.049
SA	33.94	43.92	933	1.95	0.048*	0.223
WQ	41.37	36.70	651	-0.92	0.360	− 0.104

Note. * *p* < .05; *MR*, Mean Rank; *r*, Rank-biserial correlation

**Table 4:** Differences between education levels on response variables

	Diploma or below		Associate and bachelor		Postgraduate education		X^2^	df	*p*	η²
	MR	RS	MR	RS	MR	RS				
SMT	40.80	2164	36.60	658	30.20	182	1.62	2	0.443	− 0.005
CBTT	38.20	2026	40.90	736	20.20	241	0.21	2	0.897	− 0.024
NO	39.10	2074	41.40	746	30.70	184	1.05	2	0.591	− 0.013
DE	40.00	2121	38.80	699	30.50	183	1.01	2	0.602	− 0.013
SA	41.60	2205	33.30	599	33.20	199	2.36	2	0.306	0.005
WQ	40.50	2144	38.00	684	29.20	176	1.40	2	0.495	− 0.008

*Note*. *RS*, Rank Sum; *η²*, Eta Squared

According to Table 3, the Mann-Whitney U tests revealed narrowly significant differences (*p* = .048) with small to medium effect sizes (*r* = ± 0.22) in the NO and SA variables between men and women.

The outcomes of the Kruskal-Wallis tests indicated that there is no significant statistical difference in any of the spatial memory-related variables across the three educational levels (*p* > .05).

## Discussion

In this study, the psychometric properties of an intermediate mode of assessment for measuring VSWM in older adults were investigated. Based on the results, the SMT is regarded as a stable and consistent measure for examining the performance of VSWM. In addition, analyzing the items related to face and content indicated acceptable validity in this regard. The SMT showed significant and positive correlations with VSWM (assessed by the CBTT) in terms of concurrent validity, and in terms of construct validity, it showed significant correlations with navigation and orientation as well as distance estimation. However, there was no convergence between the wayfinding dimensions and CBTT, indicating that the two instruments measure different constructs. While there is a substantial empirical connection between VSWM and wayfinding [23, 26], no correlation was found between the laboratory mode of assessing VSWM and actual wayfinding performance. A lack of ecological validity (veridicality) can be attributed to the differences between the demands of spatial cognition in laboratories and those in real-life settings. This finding reflects the suggestion of Nadolne and Stringer [19], who argued that laboratory-based assessments may not fully capture the complexity of real-world cognitive functioning. This finding reminds us that the results related to VSWM performance achieved from an instrument lacking ecological validity cannot be generalized to real-life settings. However, the existence of similar actions with the demands of spatial cognition in daily life (content validity) and high correlation with wayfinding dimensions provided verisimilitude to the SMT.

On the other hand, it was found that the SMT has an acceptable ability to differentiate between the healthy, AD, and MCI groups. This finding aligns with previous research indicating that visuospatial memory performance varies among these groups [29]. This differentiation is crucial in the early detection and intervention of cognitive impairments and can significantly improve the quality of life for these individuals [46]. In addition, the correlation between the performance measured by such an instrument and CBTT indicates the closeness of the measured construct in both of the instruments. Thus, using SMT can eliminate concerns related to the loss of control over the assessment setting of VSWM and not reflecting real-life demands.

In the context of confirming the capability to assess VSWM using SMT, the possibility of identifying differences in spatial memory and wayfinding variables among demographic groups (i.e., gender and education) was explored as a secondary objective. The results suggested a trend of a slight difference in navigation and orientation and spatial anxiety between men and women, while no difference was observed in distance estimation and overall wayfinding. This comparison did not disclose any significant difference between the two groups for VSWM as measured by either CBTT or SMT. Furthermore, no significant difference was found among the education levels for any of the variables mentioned. As this study was not primarily intended to address this objective and the assumptions for parametric statistics were not met, the results should be approached with caution.

Differences between men and women in terms of navigation and orientation, and spatial anxiety have been demonstrated in previous studies. Consistent with the findings of Muffato et al. [35], the current study found that older women experienced more spatial anxiety than older men. Furthermore, this study found that older men, similar to the orientation attitudes noted by Maffato et al., outperformed older women in terms of navigation and orientation. The study by Davis and Veltkamp [47] also reported differences in orientation between males and females.

Differences between genders in aspects related to spatial cognition have been the subject of previous studies [36, 37, 48]. The absence of differences in certain cases in this study can be attributed to the limitations of its design. The existing evidence, which suggests a reduction in spatial anxiety and an improvement in wayfinding performance [35], as well as an enhancement in visuospatial memory performance [49] with increasing education levels, was not corroborated in this study either.

The SMT combines the laboratory precision and the accuracy of ecological assessments, making it a suitable alternative for measuring VSWM in older people. It helps distinguish normal aging processes from early stages of cognitive impairment, such as MCI. Its demonstrated good reliability and various forms of validity indicate its applicability for practical purposes. Furthermore, it allows for the examination of VSWM and wayfinding variables across different demographic groups. Therefore, the SMT not only bridges the gap between laboratory and real-world applicability but also provides insights into how spatial cognition may vary based on factors such as gender.

The results of the present study can shed light on ecological studies on spatial cognition. A wide range of experiments related to spatial cognition can be designed, and relevant questions can be reinvestigated in a new way by using the developed instrument. Moreover, the present method may also be applicable to other populations with spatial cognition problems, such as stroke patients, people with different types of dementia, and people with traumatic brain injury. Future studies could explore the utility of SMT in these populations, potentially contributing to the development of more effective diagnostic and intervention strategies.

Like many other studies, this study was not without its limitations. A primary limitation is associated with the small sample size in the MCI and AD groups, as well as the high dropout rate of the healthy sample in the second session. Recruiting a sample of elderly individuals with these conditions is always a challenge. Furthermore, retaining these samples (even the healthy individuals) for a prolonged period and across multiple sessions can be complicated. Therefore, any statement about the capacity of SMT to assess non-healthy populations should be made with extreme caution, given the available data.

The availability of normative data related to VSWM for the elderly population could aid in creating ecological tools for spatial cognition. Due to the small sample size and the lack of representation of a broader population, our current study couldn’t provide such normative data. Subsequent research could generate normative data for VSWM for this demographic, utilizing both existing and new instruments, and perform a comparative analysis between them.

Finally, the current study was not designed to investigate differences between demographic groups. For instance, the lack of control in the sampling process according to gender may have resulted in observed differences in variables related to spatial memory and wayfinding. Further studies can help clarify gender and other demographic differences in a situation close to the real world.

## Conclusion

Investigating the psychometric properties of the SMT indicates that the developed instrument can reliably and validly measure VSWM performance. In addition, the measurement of VSWM using the SMT can reflect real-life wayfinding demands. Thus, SMT can be recognized as an assessment method that is close enough to both laboratory and real-life demands for spatial cognition. Furthermore, this instrument is expected to be able to distinguish healthy elderly individuals from people with MCI and AD. However, due to the sample size of the non-healthy groups in the present study, it is necessary to avoid overestimating this capacity.

### Electronic supplementary material

Below is the link to the electronic supplementary material.

Supplementary Material 1

## Data Availability

The datasets used and/or analyzed during the current study available from the corresponding author on reasonable request.

## Abbreviations

VSWM: Visuospatial Working Memory
SMT: Spatial Memory Table
CBTT: Corsi block-tapping task
WQ: Wayfinding Questionnaire
NO: Navigation and Orientation
DE: Distance Estimation
MCI: Mild Cognitive Impairment
AD: Alzheimer’s Disease
CVR: Content Validity Ratio
CVI: Content Validity Index

## References

[1] Bronfenbrenner U (1977). Toward an experimental ecology of human development. Am Psychol.

[2] Watson AJ, Brunswik E (1958). Perception and the Representative design of psychological experiments. Philos Q.

[3] Chaytor N, Schmitter-Edgecombe M (2003). The ecological validity of neuropsychological tests: a review of the literature on everyday cognitive skills. Neuropsychol Rev.

[4] Chevignard MP, Soo C, Galvin J (2012). Ecological assessment of cognitive functions in children with acquired brain injury: a systematic review. Brain Injury.

[5] Wallisch A, Little LM, Dean E (2018). Executive function measures for children: a scoping review of ecological validity. OTJR (Thorofare N J).

[6] Andrade C (2018). Internal, external, and ecological validity in research design, conduct, and evaluation. Indian J Psychol Med.

[7] Bielak AAM, Hatt CR, Diehl M (2017). Cognitive performance in adults’ daily lives: is there a lab-life gap?. Res Hum Dev.

[8] Schmuckler MA (2001). What is ecological validity? A dimensional analysis. Infancy.

[9] Lewkowicz (2001). The Concept of Ecological Validity: what are its limitations and is it bad to be invalid?. Infancy.

[10] Draheim C, Pak R, Draheim AA et al. The role of attention control in complex real-world tasks. Psychonomic Bull Rev; 29. Epub ahead of print 2022. 10.3758/s13423-021-02052-2 10.3758/s13423-021-02052-2 PMC8853083 35167106

[11] Giannouli E, Bock O, Zijlstra W. Cognitive functioning is more closely related to real-life mobility than to laboratory-based mobility parameters. Eur J Ageing. 2018;15. 10.1007/s10433-017-0434-3. Epub ahead of print. 10.1007/s10433-017-0434-3 PMC5840094 29531515

[12] Holleman GA, Hooge ITC, Kemner C, et al. The ‘Real-World Approach’ and its problems: a critique of the term ecological validity. Front Psychol. 2020;11. 10.3389/fpsyg.2020.00721. Epub ahead of print. 10.3389/fpsyg.2020.00721 PMC7204431 32425850

[13] Kazemitabar M, Kheirkhah M, Mokarrami M (2022). Does auditory attentional bias determine craving for methamphetamine? A pilot study using a word recognition dichotic listening task. Heliyon.

[14] Proulx MJ, Todorov OS, Aiken AT (2016). Where am I? Who am I? The relation between spatial cognition, social cognition and individual differences in the built environment. Front Psychol.

[15] Ponds RWHM, Hendriks M (2006). Cognitive rehabilitation of memory problems in patients with epilepsy. Seizure.

[16] Wilson BA (2008). Neuropsychological rehabilitation. Ann Rev Clin Psychol.

[17] Johnson E, Adamo-Villani N (2010). A study of the effects of immersion on short-term spatial memory. World Acad Sci Eng Technol.

[18] Diaz-Orueta U, Rogers BM, Blanco-Campal A et al. The challenge of neuropsychological assessment of visual/visuo-spatial memory: a critical, historical review, and lessons for the present and future. Front Psychol; 13. Epub ahead of print 2022. 10.3389/fpsyg.2022.962025 10.3389/fpsyg.2022.962025 PMC9447442 36081731

[19] Nadolne MJ, Stringer AY (2001). Ecologic validity in neuropsychological assessment: prediction of wayfinding. J Int Neuropsychol Soc.

[20] Amico G, Schaefer S. Negative Effects of Embodiment in a Visuo-Spatial Working Memory Task in Children, Young Adults, and Older Adults. *Front Psychol*; 12. Epub ahead of print 13 September 2021. 10.3389/fpsyg.2021.688174 10.3389/fpsyg.2021.688174 PMC8473613 34589020

[21] Popescu ST, Wexler M (2012). Spontaneous body movements in spatial cognition. Front Psychol.

[22] Colby CL. Spatial cognition. Encyclopedia of Neuroscience. Academic; 2009. pp. 165–71.

[23] Hund AM (2016). Visuospatial working memory facilitates indoor wayfinding and direction giving. J Environ Psychol.

[24] Nori R, Grandicelli S, Giusberti F (2009). Individual differences in visuo-spatial working memory and real-world wayfinding. Swiss J Psychol.

[25] Brown LA, Brockmole JR, Gow AJ et al. Processing speed and visuospatial executive function predict visual working memory ability in older adults. Exp Aging Res; 38. Epub ahead of print 2012. 10.1080/0361073X.2012.636722 10.1080/0361073X.2012.636722 22224947

[26] Mitolo M, Gardini S, Caffarra P, et al. Relationship between spatial ability, visuospatial working memory and self-assessed spatial orientation ability: a study in older adults. Cogn Process. 2015;16. 10.1007/s10339-015-0647-3. Epub ahead of print. 10.1007/s10339-015-0647-3 25739724

[27] Swanson HL. Verbal and visual-spatial working memory: what develops over a life Span? Dev Psychol. 2017;53. 10.1037/dev0000291. Epub ahead of print. 10.1037/dev0000291 28459277

[28] Priester MA, Browne T, Iachini A (2016). Treatment Access barriers and disparities among individuals with Co-occurring Mental Health and Substance Use disorders: an integrative literature review. J Subst Abuse Treat.

[29] Iachini T, Iavarone A, Senese V, et al. Visuospatial Memory in Healthy Elderly, AD and MCI: a review. Curr Aging Sci. 2012;2. 10.2174/1874612810902010043. Epub ahead of print. 10.2174/1874609810902010043 20021398

[30] Fanuel L, Plancher G, Piolino P (2020). Using more ecological paradigms to investigate working memory: strengths, limitations and recommendations. Front Hum Neurosci.

[31] Rajsic J, Wilson DE. Asymmetrical access to color and location in visual working memory. *Atten Percept Psychophys*; 76. Epub ahead of print 2014. 10.3758/s13414-014-0723-2 10.3758/s13414-014-0723-2 25190322

[32] Reppa I, Williams KE, Greville WJ (2020). The relative contribution of shape and colour to object memory. Mem Cognit.

[33] Wood JN. When do spatial and visual working memory interact? *Atten Percept Psychophys*; 73. Epub ahead of print 2011. 10.3758/s13414-010-0048-8 10.3758/s13414-010-0048-8 21264717

[34] Logie RH. *Visuo-spatial Working Memory*. Psychology. Epub ahead of print 18 March 2014. 10.4324/9781315804743

[35] Muffato V, Miola L, Pazzaglia F et al. Trajectories across the healthy adult lifespan on sense of direction, spatial anxiety, and attitude in exploring places. Front Psychol; 14. Epub ahead of print 8 August 2023. 10.3389/fpsyg.2023.1240873 10.3389/fpsyg.2023.1240873 PMC10442537 37614484

[36] Fricke M, Morawietz C, Wunderlich A et al. Successful wayfinding in age: A scoping review on spatial navigation training in healthy older adults. *Front Psychol*; 13. Epub ahead of print 16 August 2022. 10.3389/fpsyg.2022.867987 10.3389/fpsyg.2022.867987 PMC9424919 36051192

[37] Barel E, Tzischinsky O (2018). Age and sex differences in Verbal and Visuospatial abilities. Adv Cogn Psychol.

[38] Erdfelder E, FAul F, Buchner A (2009). Statistical power analyses using G*Power 3.1: tests for correlation and regression analyses. Behav Res Methods.

[39] de Rooij NK, Claessen MHG, van der Ham IJM (2019). The Wayfinding Questionnaire: a clinically useful self-report instrument to identify navigation complaints in stroke patients. Neuropsychol Rehabil.

[40] Kessels RPC, Van Zandvoort MJE, Postma A (2000). The Corsi Block-Tapping Task: standardization and normative data. Appl Neuropsychol.

[41] Mueller ST, Piper BJ (2014). The psychology experiment Building Language (PEBL) and PEBL test battery. J Neurosci Methods.

[42] Saggino A, Balsamo M, Grieco A (2004). Corsi’s Block-Tapping Task: standardization and location in factor space with the Wais–R for two normal samples of older adults. Percept Mot Skills.

[43] Leedy PD, Ormrod JE, Cape A et al. *Practical Research*, https://josemartimast.net/wp-content/uploads/2021/07/AP-Capstone-Research-Planning-and-Designing-E-Book.pdf (2010, accessed 20 August 2022).

[44] Zamanzadeh V, Ghahramanian A, Rassouli M (2015). Design and implementation content validity study: development of an instrument for measuring patient-centered communication. J Caring Sci.

[45] Steinke A, Kopp B. RELEX: an Excel-based software tool for sampling split-half reliability coefficients. Methods Psychol; 2. Epub ahead of print 2020. 10.1016/j.metip.2020.100023

[46] Cornelis E, Gorus E, Beyer I et al. Early diagnosis of mild cognitive impairment and mild dementia through basic and instrumental activities of daily living: development of a new evaluation tool. PLoS Med; 14. Epub ahead of print 2017. 10.1371/journal.pmed.1002250 10.1371/journal.pmed.1002250 PMC5349421 28291801

[47] Davis R, Veltkamp A (2020). Wayfinding strategies and spatial anxiety in older adults with and without Alzheimer’s Disease. Res Gerontol Nurs.

[48] González-Garrido AA, Gómez-Velázquez FR, Sequeira H et al. Gender Differences in Visuospatial Working Memory —Does Emotion Matter? *Int J Psychol Stud*; 5. Epub ahead of print 20 February 2013. 10.5539/ijps.v5n1p11

[49] Zarantonello L, Schiff S, Amodio P (2020). The effect of age, educational level, gender and cognitive reserve on visuospatial working memory performance across adult life span. Aging Neuropsychol Cognition.

